# Novel Molecular Insights and Evolution of Less Toxic Therapeutic Strategies in Burkitt Lymphoma

**DOI:** 10.3390/cancers17203372

**Published:** 2025-10-18

**Authors:** Coen J. Lap, Kieron Dunleavy

**Affiliations:** 1 Lombardi Comprehensive Cancer Center, Georgetown University Hospital, Washington, DC 20007, USA; coen.j.lap@medstar.net; 2 Department of Hematology and Oncology, Georgetown University Hospital, Washington, DC 20007, USA

**Keywords:** BL, MYC, EBV, risk-adapted approach, reduced-intensity regimen, relapsed disease

## Abstract

**Simple Summary:**

Burkitt lymphoma (BL) is an aggressive cancer affecting B-lymphocytes that is fast-growing but, at the same time, also highly sensitive to treatment with chemotherapy. Although most children and adolescents are cured with dose-intensive chemotherapy regimens, older patients often cannot tolerate these treatments, leading to worse outcomes. Recent studies have demonstrated that less intensive regimens are just as effective, though with fewer side effects. However, challenges remain for patients with relapsed or refractory disease and those with central nervous system involvement, as current treatment options are often ineffective. New therapeutic options, such as CAR T-cell therapy and bispecific antibodies, are currently being evaluated and offer hope in terms of improving future outcomes. This review describes the molecular biology of BL and summarizes therapeutic advances.

**Abstract:**

Burkitt lymphoma (BL) is a rare, aggressive B-cell lymphoma that is characterized by rapid tumor proliferation and frequent extra-nodal involvement. While prompt diagnosis and initiation of highly intensive chemotherapy results in cure rates over 90% in children and adolescents, outcomes in adults are more modest, as comorbidities and advancing age may compromise treatment tolerability. In recent years, intermediate-intensity regimens have been developed for BL. These are highly effective in patients of all ages and associated with significantly less treatment-related toxicity compared to traditional high-dose chemotherapy. This was demonstrated in a recent randomized study of dose-intensive R-CODOX-M/R-IVAC compared to the reduced-intensity DA-EPOCH-R regimen, which was associated with equivalent outcomes but with significantly fewer side effects. Regardless of the chemotherapy platform, CNS involvement at baseline predicts a significantly inferior outcome, and the development of an optimal approach for these patients is an area of unmet need in BL therapeutics. Patients with relapsed or refractory disease following frontline therapy have very short survival times, as currently available salvage options are largely ineffective. In this regard, novel agents such as anti-CD19 CAR-T cells and bi-specific antibodies are under development in BL. It is hoped that progress in novel drug development, alongside improved understanding of BL biology, to further elucidate its genetic and epigenetic vulnerabilities, will lead to improved outcomes for patients in the future.

## 1. Introduction

Burkitt lymphoma (BL) is a highly aggressive B-cell lymphoma characterized by rapid tumor growth that is uniformly fatal if not recognized early and treated promptly. It was first described in 1958 by Dr. Denis P. Burkitt as a fast-growing round-cell sarcoma of the jaw affecting Ugandan children but was later confirmed to be a distinct form of non-Hodgkin lymphoma (NHL) [1]. Although it is the most common childhood cancer in areas endemic for *Plasmodium falciparum*, it only accounts for 1–2% of all NHL diagnoses in adults worldwide [2,3,4]. Historically, BL has been classified into three clinical variants based on geographic location and the presence of an immunocompromised state. Moreover, the most recent classification of lymphoid neoplasms from the World Health Organization (WHO-HAEM5) recommends a further stratification based on EBV positivity status, considering this is a relevant biological subdivision based on the observed genetic and molecular distinctions between EBV-positive and EBV-negative tumors [5]. In both EBV-positive and EBV-negative BL, the translocation of the MYC oncogene is a key oncogenic event in the development of the disease and identified in virtually all cases [6,7]. Additional oncogenic mechanisms identified during the last decade further cooperate with deregulated MYC in driving BL pathogenesis [8,9].

Highly dose-intensive combination chemotherapy regimens cure over 90% of children and adolescents with BL [10]. Although adults have historically received these highly dose-intensive regimens, outcomes have been inferior to those of younger patients due to poor tolerability because of comorbidities and advancing age. Reduced-intensity regimens have been developed as an effective and less toxic alternative for adult patients with BL [11]. A recent HOVON/SAKK trial compared a high-intensity regimen (R-CODOX-M/R-IVAC) with a reduced-intensity regimen (DA-EPOCH-R) for the frontline treatment of patients with high-risk BL [12]. Although the trial stopped early, the available data did not demonstrate that either regimen was superior in terms of effectiveness. However, the reduced-intensity regimen resulted in significantly fewer toxic side effects and less need for supportive care. To further optimize outcomes and limit toxicity, other strategies have been investigated, including risk-adapted approaches that aim to tailor treatment intensity based on individual risk factors [13]. Secondary central nervous system (CNS) involvement is present in 5–10% of patients at baseline and remains a therapeutic challenge that is difficult to cure with standard frontline approaches [13,14]. Although patients with relapsed or refractory (R/R) BL have a poor prognosis, recent data has shown activity of CAR-T cell therapy and bispecific antibodies in this setting [15].

Here, we will review recent insights into the molecular biology of BL and summarize therapeutic advances with less toxic platforms and novel agents.

## 2. Epidemiology and Clinical Presentation

The World Health Organization (WHO) and the International Consensus Classification (ICC) of lymphoid neoplasms categorize BL into three epidemiological variants (Table 1) [5,16,17]. Although they share morphological and immunophenotypic features, the three variants differ in geographic prevalence, risk factors, and clinical presentation [18]. The endemic variant of BL (eBL) is observed primarily in the equatorial region of sub-Saharan Africa and Papua New Guinea, which overlaps with *Plasmodium falciparum* endemicity [2,3]. In those regions, eBL is the most common pediatric cancer and mainly affects young children with a median age of 4–7 years [19]. Although patients characteristically present with tumors of the jaw and peri-orbital region, in recent years, a change in pattern has been observed, with more frequent observation of primary abdominal tumors, including of the ileum, cecum, mesentery, and gonads [20,21]. The sporadic variant of BL (sBL) accounts for 1–2% of all non-Hodgkin lymphoma diagnoses in North America and Europe [22,23]. It is a rare lymphoma with a bimodal incidence pattern. Although most cases occur in adolescents and young adults, a second peak in incidence is observed around 70–75 years of age. Patients often present with extensive abdominal disease, especially of the ileo-cecal region. Extra-nodal involvement is common and includes the involvement of the gonads, kidneys, bone marrow, and skin. The immunodeficiency-related variant of BL (ID-BL) is most frequently seen in patients with HIV but can also occur in allograft recipients and patients with congenital immunodeficiency [24]. BL accounts for up to 35% of HIV-associated lymphomas and can develop in patients regardless of CD4 count. Even more so, the majority of patients with HIV-associated BL have higher CD4 counts (>200 cell/mm^3^), and it is assumed that cumulative HIV viremia is a more important risk factor [25,26,27]. In contrast to eBL and sBL, extra-nodal involvement in ID-BL is less common and often patients present with extensive nodal abdominal disease. Nonetheless, bone marrow and CNS involvement are frequently seen as well [28].

Staging follows the Lugano classification [29]. Because of the high incidence of bone marrow and CNS involvement at diagnosis, all patients should undergo CNS and bone marrow evaluation at presentation. The presence of more than 25% bone marrow involvement and marked leukocytosis does not preclude a diagnosis of BL, and this finding should be considered advanced disease [5]. The Burkitt Lymphoma International Prognostic Index (BL-IPI) serves as a robust prognostic tool, stratifying patients with newly diagnosed BL into three distinct risk categories (low, intermediate, and high) based on age ≥ 40 years, ECOG performance score ≥ 2, serum LDH > 3 upper limit of normal, and presence of CNS involvement as equally weighted factors [30]. Patients with zero factors are considered low-risk, those with one factor considered intermediate-risk and those with two or more factors considered high-risk. In the derivation cohort of 633 patients, 3-year progression-free survival (3-PFS) rates were 92%, 72%, and 53% (*p* < 0.0001), and 3-year overall survival (3-OS) rates were 96%, 76%, and 59% (*p* < 0.0001). Validation in an independent cohort of 457 patients confirmed these findings, establishing the index as an independent predictor of outcomes, irrespective of HIV status, disease stage, and administered first-line chemotherapy.

## 3. Pathology and Immunophenotype of BL

Although BL shares features with other aggressive B-cell lymphomas—specifically, blastic mantle cell lymphoma, diffuse large B-cell lymphoma (DLBCL), and high-grade B-cell lymphoma (HGBCL), distinguishing them remains critical, as these diseases require different management strategies [31,32,33]. Diagnosis is based on the integration of morphological findings, immunophenotypic results, and cytogenetic features, and careful consideration of each is needed to obtain an accurate diagnosis.

All three variants of BL have a similar morphology characterized by a monomorphic population of medium-sized lymphoid cells with round nuclei with prominent nucleoli, deep basophilic cytoplasm, and numerous intracytoplasmic lipid-laden vacuoles (Table 1) [34]. The tumor cells have a very high mitotic index, with a Ki-67 rate often ≥95%. The high proliferation rate is accompanied by a high rate of apoptosis. Scattered tingible body macrophages engulfing apoptotic debris among a background of darker neoplastic cells result in the characteristic “starry sky pattern”. Although a “starry sky pattern” is commonly observed in BL, it needs to be emphasized that it is not unique to BL and can also be seen in other aggressive lymphomas with a high proliferation index, including DLBCL and HGBCL [35]. Immunophenotypically, the neoplastic cells in BL typically express B-cell antigens CD19, CD20, CD22, and CD79a, as well as surface immunoglobulin—commonly IgM—with light-chain restriction [36]. Germinal center markers CD10, BCL6, and CD38 are usually positive, while MUM1 is negative. MUM1 positivity, if present, has been associated with worse outcomes [37]. The tumor cells are negative for CD5, CD23, and usually BCL-2. In approximately 20% of cases, BCL2 is found to be positive. TdT positivity should prompt consideration for a diagnosis of B-lymphoblastic lymphoma/leukemia [5]. EBV is identified in almost all cases of eBL but is only detected in 25–40% of patients with ID-BL and 10–30% of patients with sBL [38,39]. EBV-associated BL expresses CD21 [40].

## 4. Molecular Biology of BL

After the initial description of BL, the observation of herpetic-like intracellular particles in tumor cells from children with BL by virologists Michael A. Epstein and Yvonne M. Barr eventually resulted in the discovery of the eponymous Epstein–Bar virus (EBV) in 1964 [1,41]. Despite this observation over six decades ago, questions remain with respect to how EBV contributes to BL pathogenesis [42]. Various studies have shown the ability of EBV to transform B-lymphocytes in vitro and that, in humans, EBV infection precedes malignant transformation [43,44]. However, most BL cells only express a latent viral protein—namely, EBV nuclear antigen 1 (EBNA1)—as well as two non-coding RNAs (EBER), which have not been identified as oncogenic [45]. A recurrent observation in studies is the ability of EBV to promote resistance to apoptosis if triggers arise, even in the absence of latent proteins [46]. The cause of this is multifactorial and includes the modulation of BCL2 expression, interfering with caspase activation and inhibiting PKR activity [47,48,49]. New insights over the last decade suggest that deregulation of activation-induced cytidine deaminase (AID) expression has a crucial role in the development of BL. In a large study, an integrative genomic and transcriptomic analysis of 106 tumor samples from patients with HIV-negative BL (eBL and sBL) was performed [50]. This showed that in comparison to EBV-negative tumors, EBV-positive tumors were found to have significantly higher AID expression, resulting in a genome-wide increase in mutational burden. Although tumor samples from patients with eBL had significantly higher AID expression, samples from patients with sBL were also found to be associated with increased levels of AID. AID is expressed in germinal centers of lymphocytes and is crucial for normal B-cell processes such as somatic hypermutation and class-switch recombination [51]. Increased AID activity can promote double-strand break formation, resulting in translocations and mutations [52,53]. Importantly, in addition to targeting immunoglobulin (Ig) loci, AID can also induce breaks in oncogenes such as MYC [54]. Interestingly, EBV is not the only inducer of AID, and *Plasmodium falciparum* infection has been shown to be capable of this as well, suggesting that EBV and *Plasmodium falciparum* have, as expected, cooperating roles in lymphomagenesis [55,56].

The MYC family of proto-oncogenes coordinates crucial cellular processes, including proliferation, growth, cell-cycle regulation, and survival [57]. Although normally under strict transcriptional and translational control, MYC overexpression is common in human malignancies [58]. A defining feature of BL is the activation of the MYC oncogene through reciprocal translocations that juxtapose MYC located on chromosome 8q24 to any of the three immunoglobulin (IG) loci [6,7,59,60]. In over 80% of cases, the translocation partner involves the IG heavy chain (IGH) locus located on chromosome 14q32. Less frequently, MYC translocates to the IG κ (2p12) or IG λ (22q11) light-chain loci [61,62]. The placement of MYC under the direct regulation of IG enhancers results in strong constitutive activation. The chromosome breakpoints in MYC and IGH are not random, and prior research has classified breakpoint patterns induced by AID as either immediately upstream, distally upstream, or downstream of the MYC gene [63,64]. While breakpoints in eBL are frequently identified upstream of MYC and are thought to occur during somatic hypermutation, breakpoints in sBL are more frequently identified downstream of MYC as a result of aberrant resolution of double-strand breaks caused by class-switch recombination [65]. Not only does this confirm differences in pathogenesis between the epidemiological variants, but it also explains why variation exists in the prevalence of IG partners. While the presence of a MYC rearrangement is the hallmark of BL, other aggressive B-cell lymphomas can also have MYC rearrangements [66,67]. However, unlike in BL, MYC rearrangements in other aggressive B-cell lymphomas do not usually involve the IGH gene and are more frequently the result of rearrangements with either IG k, IG λ, or non-IG genes [68,69]. In addition, while the MYC rearrangement in BL commonly occurs in the background of a simple karyotype, MYC rearrangements in other lymphomas often have additional chromosomal rearrangements and abnormalities [70]. Although the 2017 revision of the WHO classification included a provisional entity of Burkitt-like lymphoma with 11q aberration, which characteristically lacks an MYC rearrangement, the 2022 update has reclassified this entity as HGBCL with 11q aberration [5,71].

**Figure 1:**
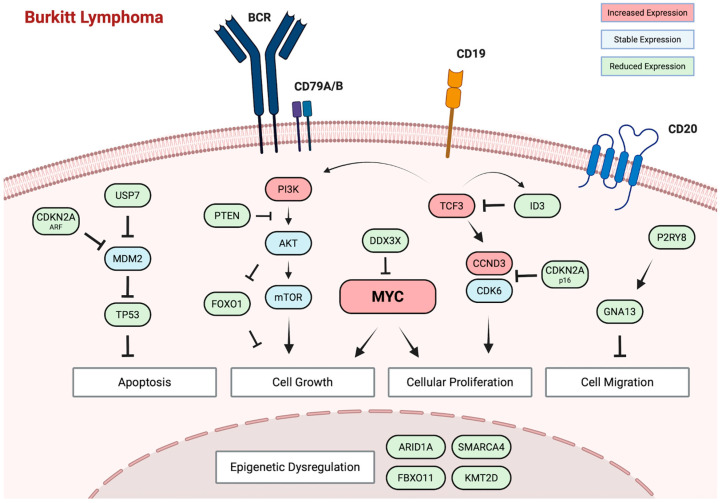
Molecular pathogenesis of Burkitt lymphoma. Central to BL pathogenesis is the deregulation of the MYC oncogene as a result of reciprocal translocation with any of the three immunoglobulin loci. Studies have implicated several recurrently mutated genes and signaling pathways that cooperate with deregulated MYC, including BCR/PI3K signaling, cell-cycle progression, epigenetic regulation, cell migration, and apoptotic pathways.

While deregulated MYC has a central role in BL pathogenesis, transgenic mice expressing MYC in B-lymphocytes (Eμ-Myc) invariably develop lymphoblastic lymphomas but not BL unless other genetic abnormalities are present [72]. Whole-genome and transcriptome sequencing analysis of all three variants of BL have implicated several recurrently mutated genes and signaling pathways that are thought to cooperate with deregulated MYC in BL pathogenesis [8,9,73]. In all variants, a crucial role is recognized for the activation of the B-cell receptor (BCR)-dependent PI3K signaling pathway (Figure 1). Recurrent mutations affecting transcription factor *TCF3* or its negative regulator, *ID3,* are observed in 30–40% of eBL tumors and approximately 70% of sBL and ID-BL tumors (Table 1). TCF3 controls BCR/PI3K signaling in BL tumor cells, enhancing continuous BCR signaling [74]. In addition, TCF3 directly activates downstream CCND3, which is also found to be mutated in over 50% of sBL and ID-BL tumors, though rarely in eBL. CCND3 drives cell-cycle progression through CDK6, which promotes cellular proliferation. Although under normal circumstances, p16 inhibits CDK6, recurrent mutations are observed in *CDKN2A*, the gene encoding p16, in up to 10% of tumors [75]. The combination of tonic BCR/PI3K signaling and uncontrolled cell-cycle progression through CDK6 supports MYC in driving uncontrolled proliferation and growth in BL.

MYC overexpression would normally induce apoptosis, yet BL cells exhibit remarkable resistance to programmed cell death [76]. While the persistence of EBV has been identified as an important factor, impaired apoptotic pathways are an additional contributor [77]. Inactivating mutations in *TP53* are identified in 15–30% of eBL tumors, up to 50% of sBL cases, and an even higher percentage in ID-BL, directly impairing p53-mediated apoptosis and cell-cycle arrest [78] Another study further supported this and revealed that in up to 55% of sBL tumors, disruption of the ARF-MDM2-p53 pathway can be observed either due to mutations in *TP53*, overexpression of MDM2, or deletion of p14ARF (*CDKN2A*) [79]. Beyond these recurrent abnormalities in the ARF-MDM2-p53 pathway, sequencing studies have implicated aberrant cell migration and epigenetic dysregulation in BL pathogenesis. Gα-13 encoded by *GNA13* is a Rho-activating G protein-coupled receptor that mediates signaling involved in cell migration, cell proliferation, and growth [80]. Mutations affecting *GNA13* and *P2RY8* have been identified across BL variants, though more frequently in sBL and ID-BL, and their loss has been associated with expansion and confinement of B-cells in germinal centers [81,82]. Recurrent mutations are also identified in genes crucial for epigenetic regulation, including *SMARCA4*, *ARID1A*, *KMT2D,* and *FBXO11*. *SMARCA4* mutations have been reported almost exclusively in sBL—in as many as 50% of cases [83]. Recent insights suggest that it is a haplo-insufficient tumor suppressor that is needed to fine-tune centrocyte cell-fate decisions by facilitating chromatin accessibility. In contrast, several studies have reported a higher frequency of *ARID1A* mutations in eBL compared to sBL. Truncating mutations in *ARID1A* are identified in up to 22% of patients with eBL and directly impair SWI/SNF nucleosome complex-mediated chromatin remodeling [84]. Although the exact role of these recurrent mutations in epigenetic regulators needs to be defined, the large number of genes impacted by mutations suggests a prominent role of epigenetic dysregulation in BL pathogenesis. Interestingly, a recent study evaluated DNA methylation patterns across 218 BL tumors [85]. Two distinct epigenetic subtypes were identified, each carrying distinct biological, transcriptomic, genomic, and clinical features not fully explained by EBV status. This all suggests an additional layer of complexity to BL pathogenesis that was not fully appreciated previously.

Side-by-side comparison reveals that differences can be observed in mutation profiles between the three epidemiological variants, which suggests differences in pathogenesis. Interestingly, the mutations identified in sBL appear to be more closely related to ID-BL than those of eBL. This observation is thought to be the result of the uniform presence of EBV in eBL tumors. Indeed, cases of sBL or ID-BL that are EBV-positive are more like eBL. Based on these insights, the latest edition of the WHO classification (WHO HAEM5) now recommends further stratification of BL based on EBV status, in addition to classification based on clinical context, as a more biologically relevant approach [5,18,42].

## 5. Management of BL in the Frontline Setting

Although most patients can be cured with intensive combination chemotherapy, given the paucity of randomized trials, the optimal therapy is controversial. Because of its high proliferation rate, BL is highly sensitive to cytotoxic chemotherapy. However, conventional chemotherapy regimens typically used for DLBCL, including CHOP, have had historically high rates of treatment failure in BL [86,87]. Most regimens developed for BL have been adopted from pediatric B-ALL protocols [88]. Regimens described in the 1980s relied on short-term rotational chemotherapy combinations with high peak drug concentrations [89]. Most of the high-intensity regimens used nowadays for the treatment of children and adults with BL have been modelled based on these multi-agent combinations, and they often include CNS-penetrating agents. However, because of the intensity of these regimens, treatment-associated toxicity and mortality are high, especially in older patients and those with comorbidities.

In 1996, Magrath and colleagues described their experience with the CODOX-M/IVAC (89-C-41) regimen that was developed for the treatment of both children and adults with BL (Table 2) [90]. Totals of 21 children and 20 adults were included, who received either three cycles of CODOX-M (*cyclophosphamide, doxorubicin, vincristine*, and *methotrexate*) if deemed to be at low risk of disease or four alternating cycles of CODOX-M and IVAC (*ifosfamide, etoposide, high-dose cytarabine*, and *IT methotrexate*) if considered high-risk. Risk stratification was based on the extent of the disease and serum LDH levels. The median age of included adults was 25 years (range, 18–56 years). No significant difference in 2-EFS was observed between children and adults (85% vs. 100%; not significant). Treatment-associated toxicities were significant, including almost universal grade 4 myelosuppression, as well as septicemia in 22.1% of patients, and profound neuropathy in 26.8%. A criticism of the study was the relatively young age of the included adults, which might have contributed to the impressive outcomes. To confirm the results and the high efficacy of the regimen, a larger prospective phase 2 study was performed in 52 adults with BL stratified, again, based on low-risk (*n* = 12) or high-risk (*n* = 40) disease [91]. The median age was 35 years (range, 15–60 years). Observed outcomes were significantly inferior, with a 2-year event-free survival (2-EFS) and 2-OS for low-risk patients of 83.3% and 81.5%, respectively, compared to 59.5% and 69.9% for those with high-risk disease. Moreover, only 80% of patients were able to complete therapy because of associated toxicities. A follow-up study attempted to curb toxicity by reducing the dose of methotrexate (3 g/m^2^) in the regimen [92]. Although treatment-associated toxicities were less frequent, a 2-PFS of 49% (95% CI 38–60%) was observed for high-risk patients, which was significantly less than previously seen, despite including patients with both BL and DLBCL in the analysis. Further refinement of the CODOX-M/IVAC regimen followed, including reductions in the doses of cyclophosphamide, vincristine, and methotrexate, but an increased dose of doxorubicin. A small phase 2 prospective trial evaluated this modified Magrath regimen in 14 patients with BL [93]. The median age was 47 years (range, 18–65 years), with 11 patients having high-risk disease. The 2-PFS for all patients was 64% (90% CI: 43–85%), and the 2-OS was 71% (90% CI: 50–91%). In high-risk patients, the 2-PFS and 2-OS were 60% (90% CI: 40–90%) and 60% (90% CI: 35–85%), respectively. Although the introduction of CD20 monoclonal antibody rituximab has resulted in significantly improved outcomes in patients with B-cell lymphomas, studies that gave looked at the incorporation of rituximab into the CODOX/M-IVAC regimen for BL have shown mixed results. In a retrospective analysis of 80 adults with BL treated with CODOX/M-IVAC with or without rituximab, a trend of improved 3-PFS (74% versus 61%) and 3-OS (77% versus 66%) was observed under rituximab-containing therapy, although results did not reach statistical significance [94]. However, subsequent prospective analyses did confirm a benefit of adding rituximab to chemotherapy. A phase 2 trial by Evens et al. evaluated the CODOX-M/IVAC regimen with the incorporation of rituximab and liposomal doxorubicin instead of doxorubicin in 25 patients with newly diagnosed BL [95]. The median age of included patients was 44 years (range, 23–70 years), and 20 patients (80%) were considered to have high-risk disease. The 2-PFS and 2-OS were 76% and 84% overall and 76% and 81% for patients with high-risk disease. Similarly, the AIDS Malignancy Consortium 048 evaluated a modified regimen of CODOX-M/IVAC with rituximab for the treatment of HIV-associated BL [96]. A total of 34 patients were enrolled who had a median age of 42 years (range, 19–55 years), among which 32 (94%) patients were considered high-risk. The reported 1-PFS was 69% (95% CI: 51–82%), and the 1-OS was 72% (95% CI: 53–84%). The 2-OS was 69% (95% CI: 50–82%). A grade 3–4 toxicity rate was observed in 79% of patients.

Besides CODOX/M-IVAC, other high-intensity regimens have been evaluated for the treatment of BL (Table 2). The hyper-CVAD regimen (*hyper-fractionated cyclophosphamide*, *vincristine*, *doxorubicin,* and *dexamethasone*) was developed at the MD Anderson Cancer Center as a modification of a similar regimen used for childhood B-ALL [98]. A total of 26 adults were enrolled in a prospective phase 2 trial who had a median age of 58 years (range, 17–79 years). A proportion of 81% of patients achieved a CR. The 3-year continuous CR rate was 61% (±11%). The 3-OS for all patients was 49% (±11%). However, subgroup analysis showed a 3-OS of 77% for patients younger than 60 years of age but only 17% for those ≥ 60 years (*p* < 0.01). Five treatment-related deaths were observed during the induction part of the trial. A follow-up study evaluated the addition of rituximab to the hyper-CVAD regimen in 31 patients with newly diagnosed BL or mature B-ALL [99]. The median age was 46 years (range, 17–77 years), with 29% of patients 60 years of age or older. CR was obtained in 86% of evaluable patients. The 3-year disease-free survival (3-DFS), 3-EFS, and 3-OS were 88%, 80%, and 89%, respectively. Although no induction deaths were reported, grade 3 to 4 myelosuppression was universal. The Cancer And Leukemia Group B (CALGB) group evaluated a regimen of multi-agent chemotherapy (CALGB9251; alternating cycles of *ifosfamide*, *methotrexate*, *vincristine*, *cytarabine*, *etoposide*, *dexamethasone* with *cyclophosphamide*, *methotrexate*, *vincristine*, *doxorubicin,* and *dexamethasone)* with or without prophylactic radiation [101] in a total of 92 patients with either BL or ALL-L3. In addition to multi-agent chemotherapy, enrolled patients received either 12 doses of intrathecal triple therapy (*methotrexate, cytarabine, dexamethasone*) with 2400cGy cranial radiation (cohort A; *n* = 52) or 6 doses of intrathecal triple therapy (cohort B; *n* = 40). The 3-EFS was not significantly different and was 52% (95% CI 38–65%) for patients in cohort A and 45% (95% CI 29–60%) for those in cohort B. Likewise, the 3-OS was not different and 54% (95% CI 40–67%) and 50% (95% CI 35–65%) for cohorts 1 and 2, respectively. However, grade 3 or higher neurological toxicity was observed in 60% of patients in cohort A versus 23% in cohort B (*p* < 0.001), resulting in an amendment of the treatment protocol to limit neurological toxicity. A treatment-associated mortality rate of 10 patients was reported. The follow-up CALGB10002 prospective trial further evaluated the same high-intensity regimen but included rituximab [102]. A total of 105 patients with BL were enrolled who had a median age of 43 years (range, 19–79 years). A proportion of 26% of patients were ≥60 years of age. The 2-EFS and 2-OS were 79% (95% CI: 66–88%) and 81% (95% CI; 68–89%), respectively. Seven patients in the study died because of treatment-associated toxicities.

In addition to these high-intensity regimens developed by groups in North America, European groups have evaluated similar multi-agent regimens adapted from pediatric ALL protocols. A French group evaluated a pediatric French Lymphoma Malin B protocol in 72 adults with BL (LMB95) [97]. Patients were classified as either low-risk (group A; resected stage I and abdominal stage II), intermediate-risk (Group B; not low or high-risk) or high-risk (Group C; CNS or ≥70% bone marrow involvement). Group A received multi-agent chemotherapy with COPAD (*cyclophosphamide*, *doxorubicin*, *vincristine*, and *prednisone*). Group B and C received a pre-phase treatment with COP (*low-dose vincristine* and *cyclophosphamide*), followed by five cycles of multi-agent chemotherapy for group B, including infusional cytarabine, methotrexate 3 g/m^2^, and intrathecal methotrexate (COPAD/CYM). Group C received eight cycles of multi-agent chemotherapy, including high-dose cytarabine, high-dose methotrexate at 8 g/m^2^, and intrathecal triple therapy (COPAD/CYVE). The 2-EFS and 2-OS were 65% (95% CI: 54–77%) and 70% (95% CI: 59–81%), respectively. Treatment-related mortality occurred in three patients. A follow-up phase 3 randomized trial further evaluated the COPAD/CYM and COPAD/CYVE regimens with or without rituximab [103]. A total of 124 patients receiving COPAD/CYM were randomly allocated to either rituximab (*n* = 60) or no rituximab (*n* = 64), and 136 patients receiving COPAD/CYVE were randomized to either rituximab (*n* = 70) or no rituximab (*n* = 66). Adding rituximab to multi-agent chemotherapy significantly improved EFS. The 3-EFS was 75% (95% CI: 66–82%) for those receiving rituximab and 62% (95% CI: 53–70%) for those receiving no rituximab (hazard ratio: 0.59; 95% CI: 0.38–0.94). The 3-OS was 83% (95% CI: 75–88%) and 70% (95% CI: 62–78%) (hazard ratio: 0.51; 95% CI: 0.30–0.86). Adding rituximab did not significantly affect toxicity. Further modifications of the LMB protocol have been investigated, including a recent prospective phase 2 trial (BURKIMAB14) that used a dose-reduction strategy for chemotherapy in patients ≤ 55 years of age once they achieved CR [104]. The 4-DFS in those who achieved CR was 86% (95% CI: 76–92%), and the 4-OS was 73% (95% CI 63–81%). The 4-DFS and 4-OS were found to be similar between those who received a dose reduction and those who did not, suggesting that dose de-escalation is safe in these patients. Likewise, the German multi-center BFM and GMALL evaluated the NHL2002 protocol for adult patients with BL in a large phase 2 trial [100]. The regimen consisted of six cycles of a multi-agent chemotherapy regimen (*high-dose methotrexate, high-dose cytarabine, cyclophosphamide, etoposide, ifosfamide, steroids, and triple intrathecal therapy*) with rituximab. A total of 363 patients were included, and those >55 years received a reduced regimen. The 5-PFS for the total cohort was 75% ± 3%, and the 5-OS was 80% ± 2%, though significant differences were observed between adolescents, adults, and the elderly (OS rates 90%, 84%, and 62%, respectively).

Despite excellent outcomes in adolescents and young adults with these high-intensity regimens, treatment-associated toxicity and mortality are major challenges in older patients and in those with comorbidities. To address this issue, a group at the NCI hypothesized that the duration of exposure to chemotherapy agents, above an effective threshold concentration, would be more crucial than peak concentrations for anti-tumor efficacy. Based on this hypothesis, they evaluated reduced-intensity treatment in 30 adults with BL in a prospective phase 2 trial [11]. As part of the trial, dose-adjusted EPOCH-R (*etoposide, prednisone, vincristine, cyclophosphamide, doxorubicin,* and *rituximab*) was evaluated in 19 patients with HIV-negative BL, as well as a short-course regimen of EPOCH with a double dose of rituximab (*SC-EPOCH-RR*) in 11 patients with HIV-positive BL [105]. The overall cohort had a median age of 33 years (range, 15–88 years) and was primarily composed of patients with intermediate-risk (73%) and high-risk (10%) disease. After a median follow-up for each regimen of 86 and 73 months, freedom from progression (FFP) and OS were 95% (95% CI: 75–99%) and 100% (95% CI: 82–100%) for patients treated with DA-EPOCH-R and 100% (95% CI: 72–100%) and 90% (95% CI: 60–98%) for those who received SC-EPOCH-RR. No treatment-related fatalities were observed, and toxicities for both regimens were predominantly of grade 1 or 2. The excellent results led to a multi-center phase 2 study of DA-EPOCH-R using a risk-adapted strategy [13]. A total of 113 adults with BL were enrolled across 22 centers. High-risk disease was defined as stage III or IV disease, elevated LDH, ECOG 2–4, or any tumor mass ≥ 7 cm. All patients underwent CNS evaluation at the time of diagnosis, and those with active CNS disease received extended intrathecal treatment. Patients with low-risk disease received three cycles of DA-EPOCH-R without CNS prophylaxis, and high-risk patients received six cycles of DA-EPOCH-R and CNS prophylaxis with intrathecal methotrexate or extended intrathecal treatment if leptomeningeal involvement was present. The median age of enrolled patients was 49 years (range, 18–86 years). A total of 28 patients (25%) were HIV-positive. A total of 98 patients (87%) were considered to have high-risk disease, with 29 (26%) of them having bone marrow or CNS involvement. After a median follow-up of 58.7 months, the 4-EFS and 4-OS for all 113 patients were 84.5% (95% CI: 76–90%) and 87.0% (95% CI: 79–92%), respectively. In the 98 patients with high-risk disease, the 4-EFS and 4-OS were 82.1% (95% CI: 73–89%) and 84.9% (95% CI: 76–91%). Of the 11 patients with CNS involvement at presentation, six developed disease progression or died. Five treatment-related deaths were reported, all in patients 50 years of age or older. Overall, treatment was well tolerated across patients of all ages. This regimen has also been investigated in patients with MYC-rearranged aggressive B-cell lymphoma and demonstrated high efficacy in a multi-center phase II trial [106].

**Figure 2:**
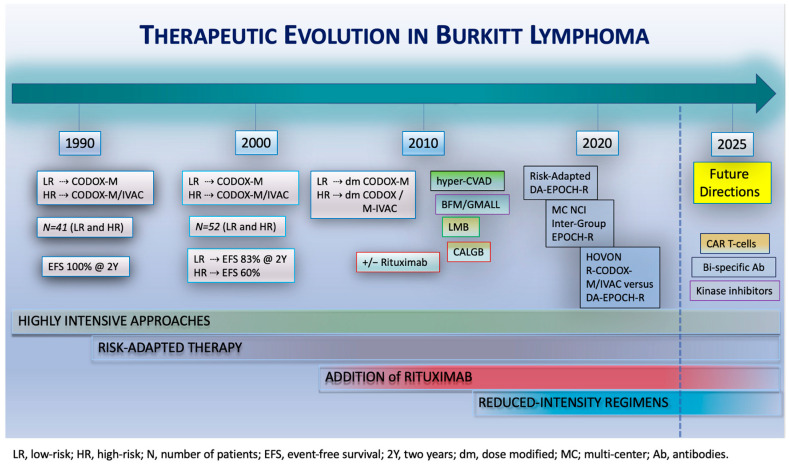
Therapeutic evolution in Burkitt lymphoma. Treatments for BL have evolved in recent decades, starting with highly intensive approaches, followed by the introduction of risk-adapted strategies and, subsequently, the addition of rituximab to chemotherapy regimens. Reduced-intensity regimens were introduced in 2013. Future studies should focus on evaluating the efficacy of novel agents, including CAR T-cell therapies, bi-specific antibodies, and kinase inhibitors.

A recent multicenter, randomized, phase 3 HOVON/SAKK trial compared a high-intensity regimen with a reduced-intensity regimen for BL [12]. Patients with a new diagnosis of high-risk BL without CNS involvement were randomized between two cycles of R-CODOX-M/R-IVAC and six cycles of DA-EPOCH-R. All patients received intrathecal CNS prophylaxis with eight doses of alternating cytarabine and methotrexate. Although the trial stopped early due to slow accrual, a total of 89 patients were enrolled. A total of 46 patients were randomized to the R-CODOX-M/R-IVAC arm and 43 to the DA-EPOCH-R arm. The median age was 52 years (IQR 37–64 years). During a median follow-up of 28.5 months (IQR: 13.2–43.7 months), no significant differences were observed in 2-PFS and 2-OS between the treatment groups. Comparison revealed a 2-PFS of 76% (95% CI: 60–86%) for patients receiving R-CODOX-M/R-IVAC and 70% (95% CI: 54–82%) for those that received DA-EPOCH-R (hazard ratio: 1.42; 95% CI: 0.64–3.18). The 2-OS was 76% for R-CODOX-M/R-IVAC and 75% for DA-EPOCH-R (hazard ratio: 1.21; 95% CI: 0.53–2.76). Despite comparable efficacy between the two regimens, reduced-intensity DA-EPOCH-R demonstrated a superior safety and tolerability profile compared to high-intensity R-CODOX-M/R-IVAC. Four patients receiving R-CODOX-M/R-IVAC went off-protocol due to excessive toxicities, whereas none of the patients treated with DA-EPOCH needed discontinuation. A high proportion of patients over 60 years old experienced grade 3–5 adverse events (90% for R-CODOX-M/R-IVAC versus 75% for DA-EPOCH-R), and patients receiving R-CODOX-M/R-IVAC developed significantly more infectious adverse events (56% R-CODOX-M/R-IVAC versus 34% DA-EPOCH-R). In addition, patients receiving R-CODOX-M/R-IVAC had significantly longer hospitalizations and required substantially more supportive care, including a significantly greater need for red blood cell and platelet transfusions. Despite the early closure of the study, it does provide valuable information and suggest that treatment with reduced-intensity DA-EPOCH-R is not inferior to high-intensity R-CODOX-M/R-IVAC treatment. However, the former regimen has significantly less toxicity and is much better tolerated by older patients and patients with comorbidities (Figure 2).

A major challenge arises when the CNS is involved at the time of diagnosis. The incidence of CNS involvement at the time of diagnosis in BL ranges between 5% and 10% [13,14,107,108]. Although most high-intensity regimens contain CNS-penetrating agents (high-dose methotrexate, cytarabine, and ifosfamide), these agents are not included in reduced-intensity regimens—notably, DA-EPOCH-R. In a multicenter study of risk-adapted DA-EPOCH-R, baseline CNS involvement was associated with an inferior outcome [13]. A recent large retrospective study further supports this and looked at outcomes of 641 adults with BL [14]. A total of 120 (19%) patients were found to have baseline CNS involvement. Those with baseline CNS involvement had a significantly lower probability of obtaining a CR than patients without baseline CNS involvement (56% versus 77%; OR: 0.45; 95% CI: 0.29–0.69). In addition, an inferior estimated 3-PFS (46% vs. 69%; HR: 2.02; 95% CI: 1.52–2.67) and estimated 3-OS (49% vs. 74%; HR: 2.18; 95% CI: 1.61–2.94) were observed. Although inferior outcomes were observed regardless of the regimen, those who received DA-EPOCH-R had particularly poor outcomes (3-PFS 25% DA-EPOCH-R versus 57% CODOX-M/IVAC versus 56% hyperCVAD). However, based on the retrospective design, it is unclear if the performance status of patients who received DA-EPOCH-R was worse at baseline. In addition to baseline CNS involvement, the retrospective study looked at CNS recurrence after contemporary first-line regimens, including DA-EPOCH-R (*n* = 146), CODOX-M/IVAC (*n* = 160), and hyperCVAD (*n* = 155). The three-year risk of CNS recurrence was significantly lower after CODOX-M/IVAC (4%) and hyperCVAD (3%) than after DA-EPOCH-R (13%) (sub-distribution hazard ratio of 3.57 DA-EPOCH-R versus others; 95% CI: 1.83–6.97). Moreover, sites of recurrence more frequently involved the CNS after DA-EPOCH-R than after high-intensity regimens (40% vs. 16%; *p* < 0.001). Not only do these findings emphasize the importance of CNS evaluation at the time of diagnosis; they also highlight the poor prognosis of these patients, regardless of frontline regimen [109]. Therefore, there remains an unmet critical need for new treatment options for patients with BL with CNS involvement.

## 6. Management of BL in the Relapsed/Refractory Setting

Despite high cure rates in the frontline setting, patients with R/R disease after frontline treatment have poor outcomes and short survival times [110,111,112]. Because of the rarity of R/R disease in BL and the exclusion of patients with BL from clinical trials evaluating new treatment options for R/R NHL, limited data are available to inform on optimal treatment strategies. In a retrospective cohort of 35 patients with R/R BL, salvage chemotherapy achieved an overall response rate (ORR) of 39%, with a median OS of 2.8 months [113]. Patients with a remission duration ≥ 6 months after frontline treatment and who achieved CR with salvage chemotherapy had superior outcomes. No salvage regimen has been demonstrated to be superior. Frequently used salvage regimens include R-ICE (*rituximab*, *ifosfamide*, *carboplatin*, and *etoposide*), R-GDP (*rituximab*, *gemcitabine*, *dexamethasone*, and *cisplatin*), rituximab with high-dose cytarabine, and R-IVAC, which should be followed by consolidation with either high-dose therapy and autologous stem-cell rescue (HDT/ASCR) or allogeneic hematopoietic stem-cell transplantation (alloSCT) if a response is obtained. Although data are limited, a small retrospective study showed a 5-PFS and 5-OS of 44% (95% CI: 21–65%) and 53% (95% CI: 34–69%), respectively, in 19 patients with R/R BL undergoing HDT/ASCR in CR2 [114]. Another retrospective analysis that included 12 patients with R/R BL found a 3-PFS and 3-OS of 36% and 37%, respectively, for patients with chemo-sensitive relapse undergoing HDT/ASCR and a 3-PFS and 3-OS of 7% for patients with chemo-resistant disease [115].

Although data regarding the efficacy of CAR T-cell therapy in R/R BL are limited, durable remissions have been reported in case reports and small case series [116,117,118]. A recent multicenter retrospective study reported real-world outcomes of CD19 CAR T-cell therapy in patients with R/R BL [119]. A total of 31 patients were included who had a median of three prior lines of therapy (range, one to six treatments). A total of 12 patients had primary refractory disease. A total of 19 patients received axicabtagene ciloleucel, 4 lisocabtagene maraleucel, 4 tisagenlecleucel, and four other agents. The 30-day ORR was 58%, with a CR rate of 41.9%. However, the 6-month CR rate was only 19.4%. The median PFS was 2.3 months (95% CI: 1.0–9.0), and the median OS was 6.0 months (95% CI: 1.9–11.5). The 28-day mortality was 16.1%. Therefore, despite excellent outcomes in R/R large B-cell lymphomas, the efficacy of CAR T-cell therapy in R/R BL has, so far, been low, with infrequent long-term disease control.

For patients with chemo-refractory disease with limited therapeutic options, bispecific antibodies can be considered [15,120]. A recent small case series reported the treatment of three patients with chemo-refractory BL, including one with secondary CNS involvement, with CD20xCD3 bispecific antibody glofitamab in combination with CD79b-targeting antibody drug conjugate polatuzumab vedotin [15]. All three patients achieved rapid CR within as few as one cycle of the combination treatment. Two patients subsequently underwent alloSCT and remained in remission for 16 and 9 months, respectively. The other patient received consolidation treatment with CAR T-cell therapy but, soon after, developed a relapse. Resumption of glofitamab resulted in repeat CR, with a current plan for consolidative alloSCT as well. These data highlight the potential of these agents, including in patients with CNS involvement, in this otherwise almost universally fatal setting, supporting future clinical trials.

## 7. Treatment Recommendations for Adult BL

BL is a highly aggressive B-cell lymphoma that requires prompt diagnosis and emergent initiation of treatment to ensure optimal outcomes. Treatment selection should not be based on the clinical variant or EBV positivity status. In patients with newly diagnosed concomitant HIV, anti-retroviral therapy should be initiated at diagnosis and continued during chemotherapy to ensure sustained viral suppression. A risk-adapted approach based on high- or low-risk disease is recommended. The recent phase 3 HOVON-SAKK trial confirmed that DA-EPOCH-R has equivalent efficacy as R-CODOX-M/R-IVAC but with significantly less toxicity and reduced need for hospitalization. In adolescents and young adults with high-risk disease, both R-CODOX-M/R-IVAC and DA-EPOCH-R can be considered as frontline treatment options. In older adults and in those with comorbidities, DA-EPOCH-R is preferred. In patients with low-risk disease, interim PET/CT imaging can identify patients for whom abbreviated therapy with three cycles of DA-EPOCH-R can be considered. Because of a high risk of CNS involvement, all patients should undergo CSF evaluation at the time of diagnosis, regardless of frontline treatment. In the absence of CNS involvement, patients with high-risk disease require CNS-directed intrathecal prophylaxis. In patients with CNS involvement, R-CODOX-M/R-IVAC might be preferred because it incorporates high-dose methotrexate. In patients receiving DA-EPOCH-R with active CNS involvement, treatment should include intrathecal or intraventricular chemotherapy as per published paradigms. For R/R patients, salvage chemotherapy followed by consolidation HDT/ASR or alloSCT can be considered or, alternatively, novel approaches such as anti-CD19 CAR-T cell therapy or bi-specific antibodies.

## 8. Future Directions and Conclusions

BL is a highly aggressive, chemo-sensitive malignancy with associated high cure rates. While intensive combination chemotherapy is highly curable in adolescents and young adults, outcomes are inferior in older adults due to treatment-associated toxicity and resultant mortality. Reduced-intensity regimens are significantly less toxic and can be administered to most adults with BL. CNS involvement is a strong predictor of poor outcomes, and for these cases, regimens incorporating CNS-penetrating agents should be used. Selecting optimal approaches in the R/R setting is challenging, but recent data obtained using novel strategies such as CAR T-cell therapy and bi-specific antibodies suggest that these approaches may be helpful, and clinical trials are ongoing. Although not many studies have evaluated the utility of circulating tumor DNA in BL, it likely has high potential as a prognostic marker and sensitive detector for minimal residual disease [121]. Improved insights into the underlying pathobiology of BL have resulted in a deeper understanding of the role of EBV in its pathogenesis, as well as the identification of distinct molecular subtypes [73]. Future studies should attempt to further identify genetic and epigenetic vulnerabilities that can be targeted with novel agents or immunotherapies. Although MYC has remained an elusive target for decades, studies have identified other potential targets in BL, including PI3K/AKT/mTOR, CDK6, and BET [122,123]. Rationally designed preclinical studies with small-molecule inhibitors targeting these proteins have revealed anti-tumor responses, but efficacy has yet to be evaluated in clinical trials. Gaining improved understanding of the potential of these agents is crucial, not only to further reduce the need for cytotoxic chemotherapy but ultimately to further improve outcomes.

## Figures and Tables

**Table 1:** Clinical and pathological features of Burkitt lymphoma variants.

	Burkitt Lymphoma		
	Sporadic BL (sBL)	Endemic BL (eBL)	Immunodeficiency-Associated BL (ID-BL)
Epidemiology	Worldwide	Plasmodium falciparum regions; specifically equatorial Africa and Papua New Guinea	Worldwide
Incidence	2–3/1,000,000 adults per year	4–5/100,000 per year	6/1000 patients with HIV
Age	Bimodal: Adolescents and AYA, ~75 years of age	Median age: 4–7 years of age	Median age: 40–45 years
Gender (M:F)	3:1	2:1	1:1
Clinical Presentation	Abdominal involvement, especially the ileocecal region, occasionally with extra-nodal disease	Jaw and peri-orbital masses. Also involves the abdomen (mesentery and retroperitoneum), gonads, and kidneys.	Nodal disease, often with extensive involvement of the GI tract and bone marrow. CD4 often >200 cells/mm^3^
CNS involvement	5–20%; leptomeningeal disease, cranial nerve palsies	<10%; cranial nerve palsies	20–30% at diagnosis
BM involvement	20–40%	10–20%	Frequent
Histopathology	Sheets of monotonous intermediate-sized cells with a “starry sky” appearance. Prominent nucleoli. Typically expressing CD19, CD20, CD22, CD79a, and PAX5 and positive for GC markers CD10 and BCL6.Commonly negative for CD5, CD23, BCL2, MUM1, and TdT. Ki-67 often >95%.		
EBV positivity	10–30%	>95%	25–45%
Chromosomal translocations	t(8;14)(q24;q32) ~80% (>endemic BL) t(2;8)(p12;q24) ~15% (>sporadic BL) t(8;22)(q24;q11) ~5% (>sporadic BL)		
Genomics	*ID3-TCF3* axis: 70–73% *CCND3:* 38–51% *TP53*: ~50% *GNA13/P2RY8*: ~25% Other recurrent mutations: *SMARCA4*, *FBXO11*, and *HIST1H2BK*	*ID3-TCF3* axis: 30–40% *CCND3:* 1.8–5% *TP53*: 15–30% *GNA13/P2RY8*: 4–6% Other recurrent mutations: *ARID1A*, *IGLL5*, *DDX3X*, and *BCL6*	*ID3-TCF3* axis: 67% *CCND3:* 67% *TP53*: 40–70% *GNA13/P2RY8*: ~25% Other recurrent mutations: *BACH2*, *IGLL5*, and *DNMT1*

BL, Burkitt lymphoma; HIV, human immunodeficiency; M, male; F, female; CNS, central nervous system; BM, bone marrow; EBV, Ebstein–Barr virus.

**Table 2:** Selected studies that have evaluated dose-intensive and reduced-intensity regimens for adults with Burkitt lymphoma.

Study	Study Type	Regimen	Disease	No. of Patients	Median Age, Range (Years)	Ann Arbor Stage 3–4	Outcome
**Dose Intensive Regimens**							
Magrath et al. [90]	Phase 2	CODOX-M/IVAC	BL/ B-ALL	20	25 (18–59)	70%	2-EFS 100%
Mead et al. [91]	Phase 2	Risk Adapted CODOX-M/IVAC	BL	52 Low-risk 12 High-risk 40	35 (15–60)	61%	Low-risk 2-EFS 83.3%, 2-OS 81.5% High-risk 2-EFS 59.5%, 2-OS 69.9%
Mead et al. [92]	Phase 2	Modified CODOX-M/IVAC	BL	53	37 (17–76)	76%	2-PFS 64% (95% CI 51–77%) 2-OS 67% (95% CI 54–80%)
Divine et al. [97]	Phase 2	Modified LMB95	BL	72	33 (18–76)	67%	2-EFS 65% (95% CI 54–77%) 2-OS 70% (95% CI 59–81%)
Thomas et al. [98]	Phase 2	HyperCVAD	BL/ B-ALL	26	58 (17–79)		3-CCR 61% (±11%) 3-OS 49% (±11%)
Thomas et al. [99]	Phase 2	HyperCVAD+ Rituximab	BL/B-ALL	31	46 (17–77)		3-EFS 80% 3-OS 89%
Evens et al. [95]	Phase 2	Modified CODOX-M/IVAC + Rituximab	BL	25 HIV+ 4 HIV− 21	44 (23–70)	80%	Overall: 2-PFS 80%, 2-OS 84% High-risk: 2-PFS 76%, 2-OS 81%
Noy et al. [96]	Phase 2	Modified CODOX-M/IVAC + Rituximab	HIV+ BL	34	42 (19–55)	74%	1-PFS 69% (95% CI 51–82%) 1-OS 72% (95% CI 53–85%)
Hoelzer et al. [100]	Phase 2 GMALL-B-ALL/NHL2002	B-NHL83 + Rituximab	BL/ B-ALL	363	42 (16–85)	71%	5-PFS 75% ± 3% 5-OS 80% ± 2%
Rizzieri et al. [101]	Phase 2	CALGB9251 ± Cranial RT	BL/ B-ALL	Cohort 1 52 Cohort 2 40	44 (18–72) 50 (17–78)	87% 93%	Cranial RT: 3-EFS 52%, 2-OS 54% No cranial RT: 3-EFS 45%, 3-OS 50%
Rizzieri et al. [102]	Phase 2	CALGB1002 + Rituximab	BL/B-ALL	105	43 (19–79)	49%	2-EFS 79% (95% CI 66–88%) 2-OS 81% (95% CI 68–89%)
Ribrag et al. [103]	Phase 3	LMB ± Rituximab	BL	257	47 (18–72)	74%	Rituximab: 3-EFS 75%, 3-OS 83% No Rituximab: 3-EFS 62%, 3-OS 70%
Ribera et al. [104]	Phase 2 Burkimab14	Modified LMB + Rituximab	BL/ B-ALL	107 HIV+ 24 HIV− 83	51 (18–80)	73%	4-DFS 86% (95% CI 76–92%) 4-OS 73% (95% CI 63–81%)
**Reduced Intensity Regimens**							
Dunleavy et al. [11]	Phase 2	DA-EPOCH-R	BL	19	25 (15–88)	58%	7-FFP 95% (95% CI 75–99%) 7-OS 100% (95% CI 82–100%)
Dunleavy et al. [105]	Phase 2	SC-EPOCH-RR	HIV+ BL	11	44 (24–60)	82%	6-FFP 100% (95% CI 72–100%) 6-OS 90% (95% CI 60–98%)
Roschewski et al. [13]	Phase 2	Risk Adapted DA-EPOCH-R	BL/ HIV+ BL	113 HIV+ 28 HIV− 85	49 (18–86)	79%	Low-risk 4-EFS 100%, 4-OS 100% High-risk 4-EFS 82.1%, 4-OS 84.9%
Chamuleau et al. [12]	Phase 3 HOVON/SAKK 127	R-CODOX-M/IVAC vs. DA-EPOCH-R	BL/ HIV+ BL	84 HIV+ 9 HIV− 75	52 (18–75)	90%	2-PFS 76% vs. 70%; HR 1.47 (95% CI 0.66–3.28) 2-OS 76% vs. 75%; HR 1.21 (95% CI 0.53–2.76)

## Data Availability

No new data were created or analyzed in this study.
